# Kinetics and prognostic value of soluble VCAM‐1 in ST‐segment elevation myocardial infarction patients

**DOI:** 10.1002/iid3.409

**Published:** 2021-02-08

**Authors:** Ahmad Hayek, Alexandre Paccalet, Laura Mechtouff, Claire C. Da Silva, Fabrice Ivanes, Hadrien Falque, Simon Leboube, Yvonne Varillon, Camille Amaz, Charles de Bourguignon, Cyril Prieur, Danka Tomasevic, Nathalie Genot, François Derimay, Eric Bonnefoy‐Cudraz, Gabriel Bidaux, Nathan Mewton, Michel Ovize, Thomas Bochaton

**Affiliations:** ^1^ Intensive Cardiological Care Division, Louis Pradel Hospital Hospices Civils de Lyon Bron France; ^2^ INSERM U1060, CarMeN Laboratory University of Lyon, Groupement Hospitalier Est Bron France; ^3^ Department of Neurology and Stroke Center, Hospices Civils de Lyon Lyon University Lyon France; ^4^ Faculty of Medicine, Loire Valley Cardiovascular Collaboration University of Tours Tours France; ^5^ Department of Cardiology and FACT CHRU de Tours Tours France; ^6^ Department of Cardiology, Louis Pradel Hospital Hospices Civils de Lyon Bron France; ^7^ Clinical Investigation Center and Heart Failure Department, Louis Pradel Hospital Hospices Civils de Lyon Bron France; ^8^ Department of Cardiovascular Functional Exploration, Louis Pradel Hospital Hospices Civils de Lyon Bron France

**Keywords:** acute coronary syndrome, cell adhesion molecules, inflammation, STEMI, VCAM‐1

## Abstract

**Background:**

Soluble vascular cell adhesion molecule‐1 (sVCAM‐1) is a biomarker of endothelial activation and inflammation. There is still controversy as to whether it can predict clinical outcome after ST‐elevation myocardial infarction (STEMI). Our aim was to assess the sVCAM‐1 kinetics and to evaluate its prognostic predictive value.

**Method:**

We prospectively enrolled 251 consecutive STEMI patients who underwent coronary revascularization in our university hospital. Blood samples were collected at admission, 4, 24, 48 h and 1 month after admission. sVCAM‐1 serum level was assessed using ELISA assay. All patients had cardiac magnetic resonance imaging at 1‐month for infarct size (IS) and left ventricular ejection fraction (LVEF) assessment. Clinical outcomes were recorded over 12 months after STEMI.

**Results:**

sVCAM‐1 levels significantly increased from admission up to 1 month and were significantly correlated with IS, LVEF, and LV end‐systolic and diastolic volume. (H48 area under curve (AUC) ≥ H48 median) were associated with an increased risk of adverse clinical events during the 12‐month follow‐up period with a hazard ratio (HR) = 2.6 (95% confidence interval [CI] of ratio = 1.2–5.6, *p* = .02). The ability of H48 AUC for sVCAM‐1 to discriminate between patients with or without the composite endpoint was evaluated using receiver operating characteristics with an AUC at 0.67 (0.57–0.78, *p* = .004). This ability was significantly superior to H48 AUC creatine kinase (*p* = .03).

**Conclusions:**

In STEMI patients, high sVCAM‐1 levels are associated with a poor clinical outcome. sVCAM‐1 is an early postmyocardial infarction biomarker and might be an interesting target for the development of future therapeutic strategies.

## Graphical abstract

A graphical presentation summarizing the putative key role of sVCAM‐1 after ST‐elevation segment MI. MI: myocardial infarction, MMP: matrix metalloproteinase, oxLDL: Oxidized Low‐Density Lipoprotein, VCAM‐1: vascular cell adhesion molecule 1.

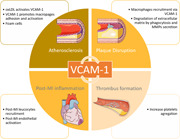

**Abbreviations**
ACS: acute coronary syndrome
CAM: cell adhesion molecule
CK: creatine Kinase
CMR: cardiac magnetic resonance
CRP: C reactive protein
IL: interleukin
IS: infarct size
LVEF: left ventricle ejection fraction
MI: myocardial infarction
STEMI: ST‐segment elevation myocardial infarction
VCAM‐1: vascular cell adhesion molecule 1

## 1 INTRODUCTION

In recent decades, new concepts of the pathogenesis of acute myocardial infarction (MI) have emerged.^1^ It is now well recognized that in the aftermath of a MI, an inflammatory response occurs and that its importance is associated with larger infarct size (IS) and increased mortality. The long‐term consequence is the onset of myocardial fibrosis and cardiac remodeling, which compromise survival and increase clinical heart failure. Leukocyte adhesion end endothelial transmigration are considered to be the main component of inflammation post‐acute coronary syndrome (ACS).^2^, ^3^ This mechanistic phenomenon is possible through cell adhesion molecules (CAM),^4^ leading to endothelial activation. In 1996, Shyu et al. reported increased levels of soluble CAM (sCAM), which are released from leukocyte and endothelial surface probably by enzymatic cleavage, in ACS patients.^5^, ^6^, ^7^

Vascular cell adhesion molecule 1 (VCAM‐1) is one of the most important CAM. VCAM‐1 plays an important role in the recruitment of inflammatory cells and thus the development of atherosclerotic plaques.^8^ Its levels have been shown to increase following ACS, and it is upregulated by tumor necrosis factor‐α and interleukin (IL)‐1β or other mediators including reactive oxygen species (ROS).^9^, ^10^, ^11^, ^12^ VCAM‐1 is a cell surface glycoprotein present in the endothelium but there is also a soluble form of VCAM‐1 (sVCAM‐1), resulting from the release of VCAM‐1 ectodomain, which is regulated by thrombin and metalloproteinase inhibitor 3 (TIMP‐3).^10^, ^13^ Several studies found sVCAM‐1 to be a predictor of cardiovascular events.^14^, ^15^ Yet, the prognostic value of sVCAM‐1 levels after ACS has come under scrutiny and there is controversy as to whether it can predict clinical outcome.^16^, ^17^, ^18^, ^19^ Lino et al. found that elevated VCAM‐1 levels were predictive of heart failure after ST‐segment elevation myocardial infarction (STEMI) while Tekin et al. showed that blood levels of sVCAM‐1 at presentation, in non‐STEMI patients, were not predictive of any adverse cardiac event.^20^, ^21^ Therefore, the objectives of our study were to describe sVCAM‐1 kinetics in patients within the first month of STEMI and to assess its prognostic value.

## 2 METHODS

### 2.1 Study design and blood sample collection

The HIBISCUS cohort is composed of consecutive patients admitted to our institution (a tertiary referral university hospital) with an acute STEMI from 2016 to 2019. Our institution Review Board and Ethics Committee approved the study. All patients gave written informed consent and the study protocol conforms to the ethical guidelines of the 1975 Declaration of Helsinki. STEMI was defined according to the European Society of Cardiology guidelines by the presence of clinical symptoms (chest pain) associated with an ST‐segment elevation of more than 2 mm in two contiguous leads on a standard 12‐lead electrocardiogram, and significant troponin‐I elevation.^22^ Urgent reperfusion was achieved in all patients by primary percutaneous intervention (PCI) at admission. All patients underwent contrast‐enhanced cardiac magnetic resonance (CMR) at 1 month after STEMI.

All individual clinical treatment and outcome data were collected prospectively in the database of the Centre for Clinical Investigation (CIC) of Hospices Civils de Lyon. Adverse clinical events were registered during follow‐up visits scheduled at 1 month, 1 year, and 2 years after the index hospitalization. A primary endpoint was defined as the composite of all‐cause death, acute MI, stroke, and rehospitalization for heart failure.

Sera from HIBISCUS patients cohort were collected at five time‐points: admission, 4 h (H4), 24 h (H24), 48 h (H48), and 1 month (M1) after reperfusion. Samples were frozen at −80°C and stored at the local hospital biobank (NeuroBioTec Biological Resource Center, Hospices Civils de Lyon). All sera from our study population were thawed only once to avoid biomarker alteration.

### 2.2 Biomarker measurement

We assessed soluble VCAM‐1 concentration using an ELISA kit (R&D systems ELISA kit). The minimum detectable dose in our conditions was 30.0 pg/ml. IL‐6 assessed using ELISA Ready‐SET‐Go (eBioscience) with a sensitivity of 2 pg/ml. Routine biomarkers were assessed in the central laboratory of the Hospices Civils de Lyon. C‐reactive protein was assessed using immunoturbidimetry (architect Abbott) (Hospices Civils de Lyon laboratory). Troponin I (Immunoassay Access1 AccuTnI Troponin I Assay) and total creatine kinase levels (Beckman Coulter Inc, expressed in IU/L) were measured at admission, at 4, 24, and 48 h after PCI. Leukocytes were collected at admission, 24, 48 h, and 1 month after admission and assessed using fluorescence‐activated cell sorting (XN‐9000 SYSMEX).

### 2.3 CMR analysis

Patients underwent CMR 1 month after admission for STEMI. All patients were scanned in a supine position using a 1.5T MAGNETOM Avanto TIM system (Siemens) as previously described.^23^ Cine free precession sequences in two‐chamber, four‐chamber, and ventricular short‐axis planes were used for quantitative ventricular measurements. Myocardial delayed enhancement sequences were assessed in short and long‐axis planes with a nonselective 180° inversion recovery 10–15 min after the injection of 0.2 mmol/kg gadolinium‐based contrast agent. IS was measured using CMRSegTools segmentation plugin (CREATIS) with OsiriX software (Pixmeo). Late gadolinium enhancement regions were quantified with a Full Width at Half Maximum algorithm and IS was expressed as a percentage of left ventricular (LV) mass. LV ejection fraction (LVEF), LV end‐diastolic volume index (LVEDVi), LV end‐systolic volume index (LVESVi), and LV mass were measured offline on all short‐axis views in the cine images (Philips View Forum, Philips Healthcare). LVEF, LVEDVi, and LVESVi assessment helped define ventricular remodeling.

### 2.4 Statistical analysis

Data are expressed as median and 95% confidence interval (CI) or interquartile range (IQR) or mean and *SD* according to their distribution. Comparisons between the different time points were performed using a paired *t*‐test with Wilcoxon matched‐paired signed‐rank test for continuous variables with the nonparametric distribution. Mann–Whitney test was used for group comparisons of continuous variables. Associations between sVCAM‐1 and clinical variables were assessed by correlation analyses (nonparametric Spearman). The ability of sVCAM‐1 to discriminate between patients with or without a clinical event was also assessed by the area under (AUC) the receiver‐operating curve (ROC). ROC curves comparison was done using DeLong et al. test. A *p* < .05 was considered significant. Multivariate analysis was performed using Cox proportional‐hazard regression. Statistical analyses were performed using GraphPad Prism 8.01. DeLong et al. test and multivariate analysis were performed using MedCalc Version 12.4.0.0.

## 3 RESULTS

### 3.1 Baseline demographics

We included 251 patients. Characteristics of the study population are presented in Table 1. Briefly, the median age was 59 ± 12 years with 79.2% male. There were 132 anterior MI (52.8%), 166 patients (66.4%) had TIMI flow grade 0–1 before and 241 (96.4%) had TIMI flow grade greater than or equal to 2 after PCI. Two hundred and sixteen patients (86.4%) were Killip 1 at admission and median LVEF at admission was 52% (interquartile range (IQR) [46–58]).

**Table 1:** Baseline characteristics of the study population

Baseline characteristics (*n* = 251 patients)	
Age, years	59 ± 12
Male sex, no (%)	199 (79.3)
BMI, kg/m^2^	26.8 ± 4.4
Hypertension, no (%)	70 (27.9)
Hypercholesterolemia, no (%)	70 (27.9)
Diabetes mellitus, no (%)	37 (14.7)
Current smoker, no (%)	126 (50.2)
Clinical characteristics	
Time from symptoms to PCI, min	200 [145–315]
Anterior MI, no (%)	132 (52.6)
Killip status = 1, no (%)	216 (86.1)
TIMI at admission = 0–1	167 (66.5)
LVEF at 1 month (%)	52 [46–58]
Biochemical analyses	
Peak troponin I, ng/L	43,904 [15,731–114,083]
Peak creatine kinase, mUI/L	1561 [686–3666]
CRP at admission, mg/L	2.6 [1.4–6.2]
Peak CRP, mg/L	17.9 [7.1–47.1]
Admission BNP, nmol/L	31 [15–80]
Admission creatinine, mmol/L	71 [61–83]
Admission hemoglobin, g/L	140 [130–150]
Leukocytes count at admission, g/L	11.8 [9.3–14.5]
Total cholesterol, g/L	1.98 [1.70–2.36]
LDL cholesterol, g/L	1.26 [1.01–1.57]
HbA1c (%)	5.7 [5.5–6.0]

*Note:* Date are expressed as mean ± *SD* or median and interquartile range.

Abbreviations: BMI, body mass index; BNP, brain natriuretic peptide; CRP, C‐reactive protein; HbA1c, glycated hemoglobin; LDL, low density lipoprotein; LVEF, left ventricular ejection fraction; TIMI, thrombolysis in myocardial infarction.

### 3.2 Soluble VCAM‐1 kinetics after STEMI

**Figure 1:**
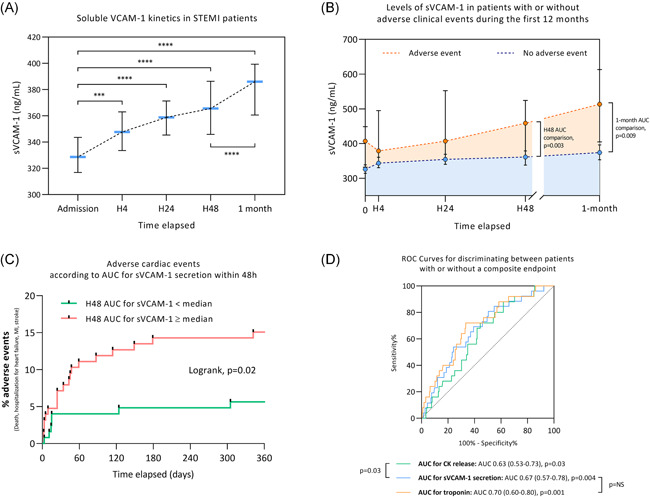
(A) sVCAM‐1 kinetics in STEMI patients within the first month (*n* = 251 patients). (B) Level of sVCAM‐1 in patients with or without adverse clinical events (all‐cause death, MI, stroke, and hospitalization for heart failure) during the first 12 months after MI (*n* = 251 patients). (C) Adverse clinical events according to AUC for sVCAM‐1 secretion within 48 h (*n* = 251 patients). (D) ROC for discriminating patients with or without a composite endpoint (adverse clinical event). ***p < .001, ****p < .0001. AUC, area under curve; CK, creatine kinase; MI, myocardial infarction; ROC, receiver operating characteristic; sVCAM‐1, soluble VCAM‐1; STEMI, ST‐segment elevation myocardial infarction

The temporal profile of sVCAM‐1 after STEMI is presented in Figure 1A, illustrating a significant and constant increase since admission up to 1 month. Median admission sVCAM‐1 level was 328.7 ng/ml (IQR [269.3–413.3]) with a continuous increase and reached 386.1 ng/ml (IQR [309.0–471.0]) at 1 month (*p* < .0001 between admission and 1‐month). sVCAM‐1 kinetics were also presented according to IS, localization of MI, and HS troponin (Figure S1). Patients with an IS higher than 25% had higher levels of sVCAM‐1 when comparing to patients with an IS lower than 25% (*p* = .03). Patients with an anterior MI had a slight tendency for higher levels of sVCAM‐1 when compared to nonanterior MI (Figure S1).

Other main inflammatory markers were also assessed. We found a rise in IL‐6 with a peak at H24 (5.5 pg/ml IQR [2.2–11.0]) and in C‐reactive protein (CRP) at H48 (18.1 mg/L IQR [7.6–52.0]). AUC sVCAM‐1 level at H48 was correlated with IL‐6 peak (*r* = .17, *p* = .01) and with CRP peak (*r* = .19, *p* = .003).

### 3.3 Soluble VCAM‐1 levels, IS, and ventricular remodeling

Wa assessed the area under curve for sVCAM‐1 within the first 48 h (H48 AUC for sVCAM‐1). We observed a significant correlation between IS (assessed by cardiac magnetic resonance imaging) and H48 AUC for sVCAM‐1 (*r* = .20, *p* = .007) (Figure S2A). We also found a significant correlation between H48 AUC for sVCAM‐1 and ventricular remodeling. H48 AUC for sVCAM‐1 was correlated with left ventricular end‐diastolic volume (LVEDV, *r* = .18, *p* = .02) and left ventricular end‐systolic volume (LVESV, *r* = .22, *p* = .003) and was inversely correlated with LVEF (*r* = −0.17, *p* = .02) (Figure S2B–D).

### 3.4 sVCAM‐1 and clinical outcomes

Twenty‐seven patients experienced an adverse clinical event during the 12‐month follow‐up period (four all‐cause deaths, six MI, three strokes, and 14 hospitalizations for heart failure). As shown in Figure 1B, The AUC for sVCAM‐1 release within the first 48 h was significantly increased in the group of patients with a clinical adverse event as compared with patients without clinical event, with a median of 1248 arbitrary units IQR [993–1491] versus 991 arbitrary units IQR [832–1225] (*p* = .003).

Furthermore, we divided the study population into two groups according to the H48 AUC for sVCAM‐1: a group with H48 AUC for sVCAM‐1 below the median value and a group with H48 AUC for sVCAM‐1 equal to or above the median value. Patients with H48 AUC for sVCAM‐1 equal to or above the median value were more likely to experiment an adverse clinical event within the 12 months of follow‐up compared to patients with H48 AUC for sVCAM‐1 below the median value with a hazard ratio (HR) = 2.6 (95% CI of ratio = 1.2–5.6, *p* = .02) (Figure 1C). The ability of H48 AUC for sVCAM‐1 to discriminate between patients with or without the composite endpoint was also evaluated. The area under the curve was assessed at 0.67 (0.57–0.78) *p* = .004. This ability to discriminate between patients with or without the composite endpoint was significantly superior to that of H48 AUC creatine kinase (*p* = .03) but not to that of H48 AUC troponin (Figure 1D).

### 3.5 Level of sVCAM‐1 is associated with adverse clinical events upon admission

**Figure 2:**
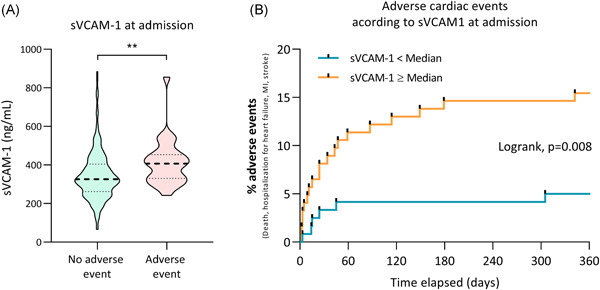
(A) Violin plot of sVCAM‐1 serum level at admission in STEMI patients. **p < .01. (B) Adverse clinical events (all‐cause death, myocardial infarction, stroke and hospitalization for heart failure) according to sVCAM‐1 at admission. STEMI, ST‐segment elevation myocardial infarction; sVCAM‐1, soluble vascular cell adhesion molecule‐1

The sVCAM‐1 level was significantly increased upon admission in the group presenting an adverse clinical event within the 12 first months, reaching 407.1 ng/ml IQR [330.0–453.7] compared to 326.1 ng/ml IQR [262.4–404.8] in the group without clinical event in the follow‐up period (*p* = .003) (Figure 2A). Patients with an sVCAM‐1 level at admission equal to or above the median value were more likely to experiment an adverse clinical event during the first 12 months of follow‐up (HR = 3.3; 95% CI of ratio = 1.5–7.1, *p* = .008) (Figure 2B). In multivariable Cox regression analyses with models including age, gender, hypertension, renal dysfunction (eGFR < 60 ml/min/1.73 m^2^), creatine kinase peak, and TIMI flow grade before PCI, sVCAM‐1 remained associated with an increased risk of experiencing the composite endpoint during the 12 months of follow‐up (adjusted HR = 2.9 [1.0–8.2], *p* = .046).

## 4 DISCUSSION

The main findings in the present study were that (1) there is a sustained endothelial activation evaluated by sVCAM‐1 release, which lasts at least one month following MI, and (2) sVCAM‐1 level upon admission is an early prognosis biomarker following STEMI, correlated with IS, cardiac remodeling, and adverse clinical events.

MI is generally the result of an atherosclerotic plaque rupture resulting in thrombus formation. CAM is involved in the thrombus formation through the coagulation pathway and platelet adhesion.^2^ Inflammation plays a pivotal and consequent role with cytokine, thrombin, and sCAM overproduction. Soluble CAM allows recruitment of circulating leukocyte cells to the inflammatory site and therefore maintains the inflammatory process.^24^ VCAM‐1 is a CAM mediating the adhesion mainly of leukocytes to the endothelium. Our study highlights the concept of endothelial activation post‐STEMI since sVCAM‐1 levels significantly increase during the first month. Our results are in accordance with previous observations that found a change in sVCAM‐1 levels related to endothelial activation and release after MI reperfusion.^25^ Mulvihill et al.^26^ found a sustained sVCAM‐1 increase up to 6 months following an ACS. Furthermore, the time course of VCAM‐1 release was evaluated by Uitterdijk et al. on swine.^27^ They reported a significant increase in sVCAM‐1 expression during the first week after MI followed by a decrease up to 1 month. We can hypothesize that this sustained activation of VCAM‐1 within the first month may participate in the LV remodeling process.

Here, we demonstrated that sVCAM‐1 is an early biomarker of severity after STEMI and is correlated with IS, LVEF, and LV remodeling. sVCAM‐1 appeared as an efficient predictor of negative clinical outcome as illustrated by the ROC curve, superior to a classic marker of IS such as creatine kinase. We already demonstrated that, besides IS, several variables contribute strongly to the clinical outcomes of STEMI patients and inflammation has probably an important role to play.^28^

**Figure 3:**
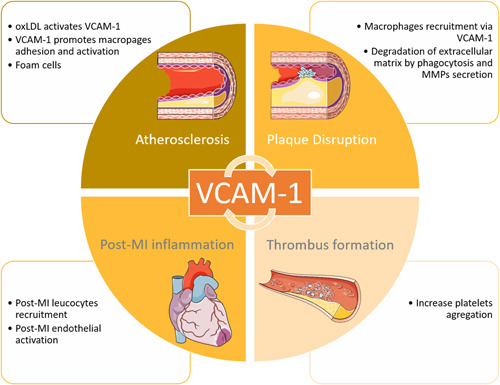
Central illustration: Graphical presentation summarizing the putative key role of sVCAM‐1 after ST‐elevation segment myocardial infarction. MI, myocardial infarction; MMP, matrix metalloproteinase; oxLDL, oxidized low‐density lipoprotein; sVCAM‐1, soluble vascular cell adhesion molecule 1

Our data highlight the importance of sVCAM‐1 at the acute phase of MI (Figure 3). Few studies on STEMI patients have been published in this setting, with the majority of them including non‐STEMI patients.^16^, ^19^, ^21^ The largest study prospectively enrolled all‐coming acute MI and showed that elevated levels of sVCAM‐1 associated with a reduced level of IL‐17A at the time of admission for acute MI were associated with an increased risk of death and recurrent MI during the first year of follow‐up, in line with our results.^18^

Soluble VCAM‐1 has been reported to be higher in patients with LV dysfunction and is associated with a reduced prognosis. In a prospective study of 48 patients with acute MI, Lino et al. found that patients with heart failure had higher values of sVCAM‐1.^20^ Postadzhiyan et al.^16^ suggested that CAM serum levels may be more powerful than other inflammatory markers in predicting increased risk for cardiovascular events in patients after ACS. IS, as well as heart failure, involves physiological and pathological pathways that lead to an increase in several biomarkers.^29^ In a clinical setting, troponin and creatinin phosphokinase are established biomarkers and correlate with IS and clinical outcomes. Our results support sVCAM‐1 as a possible early prognosis biomarker. Alongside other markers, it could play a role in the assessment and management of STEMI patients at the acute phase and in identifying patients with a higher risk in the early stage. Postadzhiyan et al.^16^ also suggested that VCAM‐1 release precedes myocardial injury and can hence identify patients with unstable atherosclerotic plaque formation even before complete microvascular obstruction.

The role of inflammation in the pathogenesis of MI leads to consider therapeutic strategies that target inflammation mediators.^30^ This has been especially the case in atherosclerosis with the use of AGI‐1067 therapy as an inhibitor of VCAM‐1 gene expression.^31^ Cybuslky et al.^32^ demonstrated that this drug significantly reduced the extent of atherosclerotic lesions in a murine model of accelerated atherosclerosis, with decreased VCAM‐1 expression. However, there is no available data regarding VCAM‐1 inhibition during MI and it might be of interest to study the effect of sVCAM‐1 inhibition in a preclinical model of MI. Also, as sVCAM‐1 plays a key role in the onset of inflammation in a MI setting with a strong correlation with poor outcomes, it raises the question whereas these patients would be good candidates for anti‐inflammatory therapeutic pathways such as colchicine and IL‐1 inhibitors that recently showed promising results.^33^, ^34^, ^35^

### 4.1 Limitation of our study

There are a couple of limitations in our study. Blood samples were collected at the acute stage of STEMI and at 1 month after. Our cohort may lack an intermediate point of sampling (e.g., 1 week) and a late point of sampling (e.g., 1 year) to assess more accurately the kinetics of sVCAM‐1 and to evaluate if it is back to baseline at 1 year. Furthermore, our study is descriptive and our data do not allow us to establish a cause‐to‐effect relationship. We assessed the soluble form of VCAM‐1 that may differ from the cell surface form. This may question the representativeness of sVCAM‐1 for endothelial activation. However, the study from Nakai et al. may help to answer this question. They showed that sVCAM‐1 was correlated with the expression of VCAM‐1 messenger RNA in patients with atherosclerotic aorta.^36^ Thus, by extrapolating their results, we suppose that sVCAM‐1 at the acute phase of MI may be representative of the cell surface form.

## 5 CONCLUSION

sVCAM‐1 serum levels increase within the first month following STEMI and are associated with IS, LV remodeling, and adverse clinical events at 1‐year. These observations are of major clinical relevance as they warrant consideration around sVCAM‐1 as an early biomarker of poor prognosis in STEMI patients.

## CONFLICT OF INTERESTS

The authors declare that there is no conflict of interests.

## AUTHOR CONTRIBUTIONS

Dr. Ahmad Hayek contributed mainly to the writing of the article. M. Alexandre Paccalet participated in the handling of the experiments and analysis of the samples. Dr. Laura Mechtouff participated in the analysis of the samples. Dr. Claire Crola Da Silva participated in the analysis of the samples. Pr Fabrice Ivanes: Participated in the proofreading of the article, corrections and proposal of additional analysis. Dr. Hadrien Falque participated in the experiments. Dr. Simon Leboube participated in the experiments. M^me^ Yvonne Varillon contributed largely to the handling of all technical aspects of the research. M^me^ Camille Amaz contributed to all statistical analysis. M. Charles de Bourguignon contributed by analysing all magnetic resonance imaging for all patients. Dr. Cyril Prieur participated largely in the patients' inclusion process. Dr. Danka Tomasevic participated largely in the patients' inclusion process. Dr. Nathalie Genot participated largely in the patients' inclusion process. Dr. François Derimay participated largely in the patients' inclusion process. Pr Eric Bonnefoy‐Cudraz participated in the design of the study and its development. Dr. Gabriel Bidaux participated in the design of the study and its development. Pr Nathan Mewton participated in the design of the study and its development. Pr Michel Ovize participated in the design of the study and its development. Dr. Thomas Bochaton participated in the design of the study and its development, contributed to the writing of the article, analysis of samples, and statistical analysis.

## Supporting information

Supporting information.

Supporting information.

Supporting information.

## Data Availability

Data are the property of the Hospices Civils de Lyon. Data can be requested by submitting an email to the person in charge at the Direction de la Recherche Clinique et de l'Innovation des Hospices Civils de Lyon: METZINGER Anne, anne.metzinger@chu-lyon.fr.

## References

[1] Libby P . Current concepts of the pathogenesis of the acute coronary syndromes. Circulation. 2001;104:365‐372. 10.1161/01.CIR.104.3.365 11457759

[2] Jang Y , Lincoff AM , Plow EF , Topol EJ . Cell adhesion molecules in coronary artery disease. J Am Coll Cardiol. 1994;24:1591‐1601. 10.1016/0735-1097(94)90162-7 7963103

[3] Libby P , Ridker PM , Maseri A . Inflammation and atherosclerosis. Circulation. 2002;105:1135‐1143. 10.1161/hc0902.104353 11877368

[4] Ross R . Atherosclerosis—an inflammatory disease. N Engl J Med. 1999;340:115‐126. 10.1056/NEJM199901143400207 9887164

[5] Shyu KG , Chang H , Lin CC , Kuan P . Circulating intercellular adhesion molecule‐1 and E‐selectin in patients with acute coronary syndrome. Chest. 1996;109:1627‐1630. 10.1378/chest.109.6.1627 8769521

[6] Pigott R , Dillon LP , Hemingway IH , Gearing AJH . Soluble forms of E‐selectin, ICAM‐1 and VCAM‐1 are present in the supernatants of cytokine activated cultured endothelial cells. Biochem Biophys Res Commun. 1992;187:584‐589. 10.1016/0006-291X(92)91234-H 1382417

[7] Gearing AJ , Hemingway I , Pigott R , Hughes J , Rees AJ , Cashman SJ . Soluble forms of vascular adhesion molecules, E‐selectin, ICAM‐1, and VCAM‐1: pathological significance. Ann NY Acad Sci. 1992;667:324‐331. 10.1111/j.1749-6632.1992.tb51633.x 1285023

[8] O'Brien KD , Allen MD , McDonald TO , et al. Vascular cell adhesion molecule‐1 is expressed in human coronary atherosclerotic plaques: implications for the mode of progression of advanced coronary atherosclerosis. J Clin Invest. 1993;92:945‐951. 10.1172/JCI116670 7688768 PMC294934

[9] Ghaisas NK , Shahi CN , Foley B , et al. Elevated levels of circulating soluble adhesion molecules in peripheral blood of patients with unstable angina. Am J Cardiol. 1997;80:617‐619. 10.1016/S0002-9149(97)00432-3 9294992

[10] Singh R , Mason J , Lidington E , et al. Cytokine stimulated vascular cell adhesion molecule‐1 (VCAM‐1) ectodomain release is regulated by TIMP‐3. Cardiovasc Res. 2005;67:39‐49. 10.1016/j.cardiores.2005.02.020 15949468

[11] Lin CC , Pan CS , Wang CY , Liu SW , Hsiao LDer , Yang CM . Tumor necrosis factor‐alpha induces VCAM‐1‐mediated inflammation via c‐Src‐dependent transactivation of EGF receptors in human cardiac fibroblasts. J Biomed Sci. 2015;22:53. 10.1186/s12929-015-0165-8 26173590 PMC4502472

[12] Cook‐Mills JM , Marchese ME , Abdala‐Valencia H . Vascular cell adhesion molecule‐1 expression and signaling during disease: regulation by reactive oxygen species and antioxidants. Antioxidants Redox Signal. 2011;15:1607‐1638. 10.1089/ars.2010.3522 PMC3151426 21050132

[13] Barthel SR , Johansson MW , Annis DS , Mosher DF . Cleavage of human 7‐domain VCAM‐1 (CD106) by thrombin. Thromb Haemost. 2006;95:873‐880. 16676080

[14] De Lemos JA , Hennekens CH , Ridker PM . Plasma concentration of soluble vascular cell adhesion molecule‐1 and subsequent cardiovascular risk. J Am Coll Cardiol. 2000;36:423‐426. 10.1016/S0735-1097(00)00742-7 10933352

[15] Mulvihill NT , Foley JB , Murphy RT , Curtin R , Crean PA , Walsh M . Risk stratification in unstable angina and non‐Q wave myocardial infarction using soluble cell adhesion molecules. Heart. 2001;85:623‐627. 10.1136/heart.85.6.623 11359739 PMC1729754

[16] Postadzhiyan AS , Tzontcheva AV , Kehayov I , Finkov B . Circulating soluble adhesion molecules ICAM‐1 and VCAM‐1 and their association with clinical outcome, troponin T and C‐reactive protein in patients with acute coronary syndromes. Clin Biochem. 2008;41:126‐133. 10.1016/j.clinbiochem.2007.09.001 18061588

[17] Harling L , Lambert J , Ashrafian H , Darzi A , Gooderham NJ , Athanasiou T . Pre‐operative serum VCAM‐1 as a biomarker of atrial fibrillation after coronary artery bypass grafting. J Cardiothorac Surg. 2017;12:70. 10.1186/s13019-017-0632-2 28821262 PMC5563046

[18] Simon T , Taleb S , Danchin N , et al. Circulating levels of interleukin‐17 and cardiovascular outcomes in patients with acute myocardial infarction. Eur Heart J. 2013;34:570‐577. 10.1093/eurheartj/ehs263 22956509

[19] Rallidis LS , Gika HI , Zolindaki MG , Xydas TA , Paravolidakis KE , Velissaridou AH . Usefulness of elevated levels of soluble vascular cell adhesion molecule‐1 in predicting in‐hospital prognosis in patients with unstable angina pectoris. Am J Cardiol. 2003;92:1195‐1197. 10.1016/j.amjcard.2003.07.029 14609595

[20] Lino DOC , Freitas IA , Meneses GC , et al. Interleukin‐6 and adhesion molecules VCAM‐1 and ICAM‐1 as biomarkers of post‐acute myocardial infarction heart failure. Brazilian J Med Biol Res. 2019;52. 10.1590/1414-431x20198658 PMC6886400 31778438

[21] Tekin G , Tekin A , Sipahi I , Kaya A , Sansoy V . Plasma concentration of soluble vascular cell adhesion molecule‐1 and oncoming cardiovascular risk in patients with unstable angina pectoris and non‐ST‐segment elevation myocardial infarction. Am J Cardiol. 2005;96:379‐381. 10.1016/j.amjcard.2005.03.080 16054462

[22] Thygesen K , Alpert JS , Kristian Thygesen C , White HD , Lane Cardiovascular Service G . Fourth universal definition of myocardial infarction (2018). Eur Heart J. 2019;40:237‐269. 10.1093/eurheartj/ehy462 30165617

[23] Bernelin H , Mewton N , Si‐Mohamed S , et al. Neprilysin levels at the acute phase of ST‐elevation myocardial infarction. Clin Cardiol. 2019;42:32‐38. 10.1002/clc.23090 30284298 PMC6436495

[24] Gearing AJH , Newman W . Circulating adhesion molecules in disease. Immunol Today. 1993;14:506‐512. 10.1016/0167-5699(93)90267-O 7506035

[25] Kerner T , Ahlers O , Reschreiter H , Bührer C , Möckel M , Gerlach H . Adhesion molecules in different treatments of acute myocardial infarction. Crit Care. 2001;5:145‐150. 10.1186/cc1014 11353931 PMC31578

[26] Mulvihill NT , Foley JB , Murphy R , Crean P , Walsh M . Evidence of prolonged inflammation in unstable angina and non‐Q wave myocardial infarction. J Am Coll Cardiol. 2000;36:1210‐1216. 10.1016/S0735-1097(00)00824-X 11028472

[27] Uitterdijk A , Groenendijk BCW , Gorsse‐Bakker C , et al. Time course of VCAM‐1 expression in reperfused myocardial infarction in swine and its relation to retention of intracoronary administered bone marrow‐derived mononuclear cells. PLOS One. 2017;12:e0178779. 10.1371/journal.pone.0178779 28628621 PMC5476248

[28] Bochaton T , Claeys MJ , Garcia‐Dorado D , et al. Importance of infarct size versus other variables for clinical outcomes after PPCI in STEMI patients. Basic Res Cardiol. 2019;115:4. 10.1007/s00395-019-0764-8 31832789

[29] Chow SL , Maisel AS , Anand I , et al. Role of biomarkers for the prevention, assessment, and management of heart failure: a scientific statement from the American Heart Association. Circulation. 2017;135:e1054‐e1091. 10.1161/CIR.0000000000000490 28446515

[30] Preiss DJ , Sattar N . Vascular cell adhesion molecule‐1: a viable therapeutic target for atherosclerosis? Int J Clin Pract. 2007;61:697‐701. 10.1111/j.1742-1241.2007.01330.x 17394445

[31] Kunsch C , Luchoomun J , Grey JY , et al. Selective inhibition of endothelial and monocyte redox‐sensitive genes by AGI‐1067: a novel antioxidant and anti‐inflammatory agent. J Pharmacol Exp Ther. 2004;308:820‐829. 10.1124/jpet.103.059733 14617690

[32] Cybulsky MI , Iiyama K , Li H , et al. A major role for VCAM‐1, but not ICAM‐1, in early atherosclerosis Find the latest version: a major role for VCAM‐1. J Clin Invest. 2001;107:1255‐1262. 10.1172/JCI11871 11375415 PMC209298

[33] Asahina A , Tada Y , Nakamura K , Tamaki K . Colchicine and griseofulvin inhibit VCAM‐1 expression on human vascular endothelial cells—evidence for the association of VCAM‐1 expression with microtubules. J Dermatol Sci. 2001;25:1‐9. 10.1016/S0923-1811(00)00097-9 11154858

[34] Nidorf SM , Fiolet ATL , Mosterd A , et al. Colchicine in patients with chronic coronary disease. N Engl J Med. 2020;383:1838‐1847. 10.1056/nejmoa2021372 32865380

[35] Ridker PM , Everett BM , Thuren T , et al. Antiinflammatory therapy with canakinumab for atherosclerotic disease. N Engl J Med. 2017;377:1119‐1131. 10.1056/nejmoa1707914 28845751

[36] Nakai K , Itoh C , Kawazoe K , et al. Concentration of soluble vascular cell adhesion molecule‐1 (VCAM‐1) correlated with expression of VCAM‐1 mRNA in the human atherosclerotic aorta. Coron Artery Dis. 1995;6:497‐502. 7551271

