# Supplementary figures and images for: Kinetics and prognostic value of soluble VCAM‐1 in ST‐segment elevation myocardial infarction patients
Source: Immun Inflamm Dis. 2021 Feb 8;9(2):493–501. doi: 10.1002/iid3.409 (PMC8127550; doi:10.1002/iid3.409)

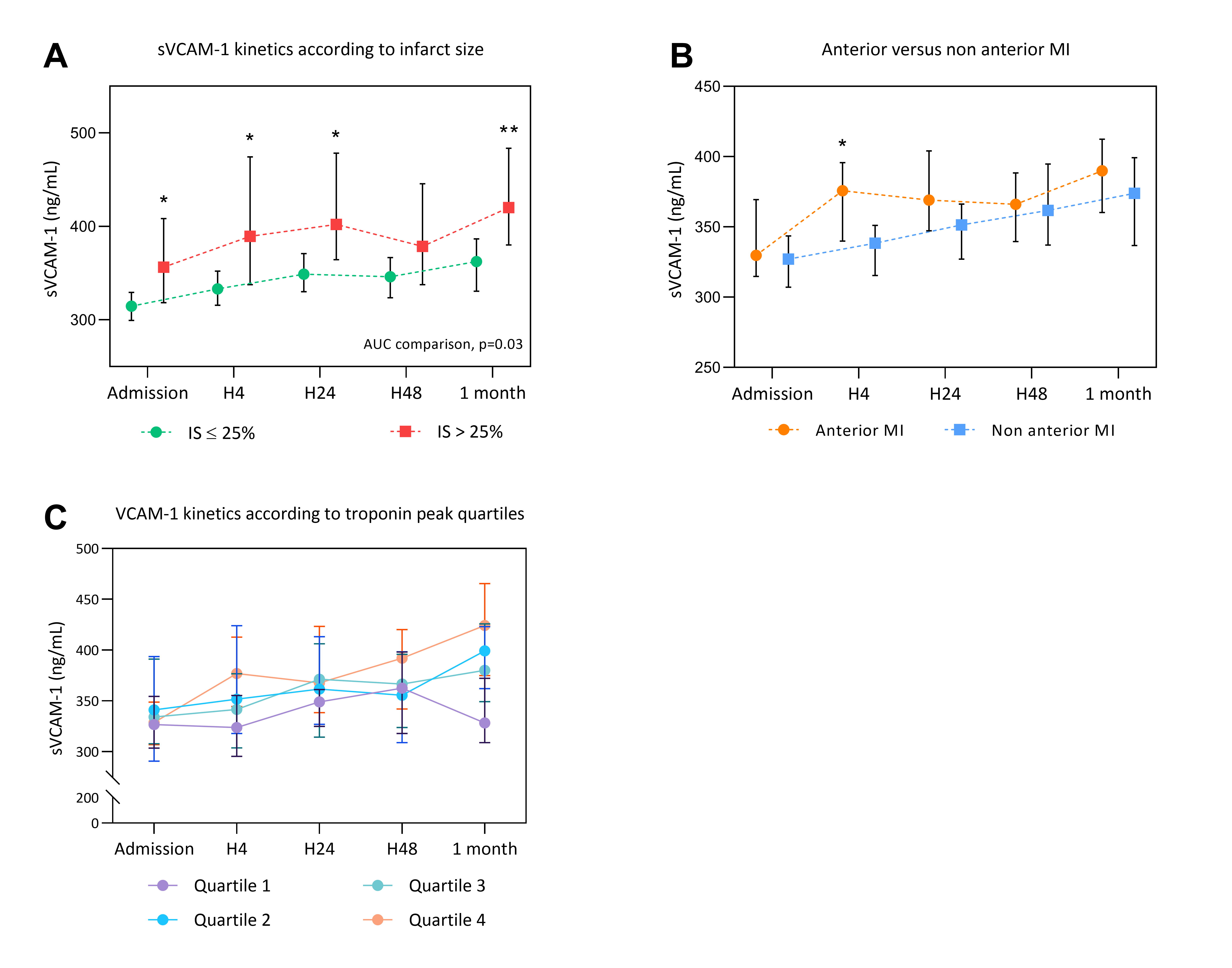

Supplement: Supplementary file 1 — Supporting information. [file IID3-9-493-s003.tif]

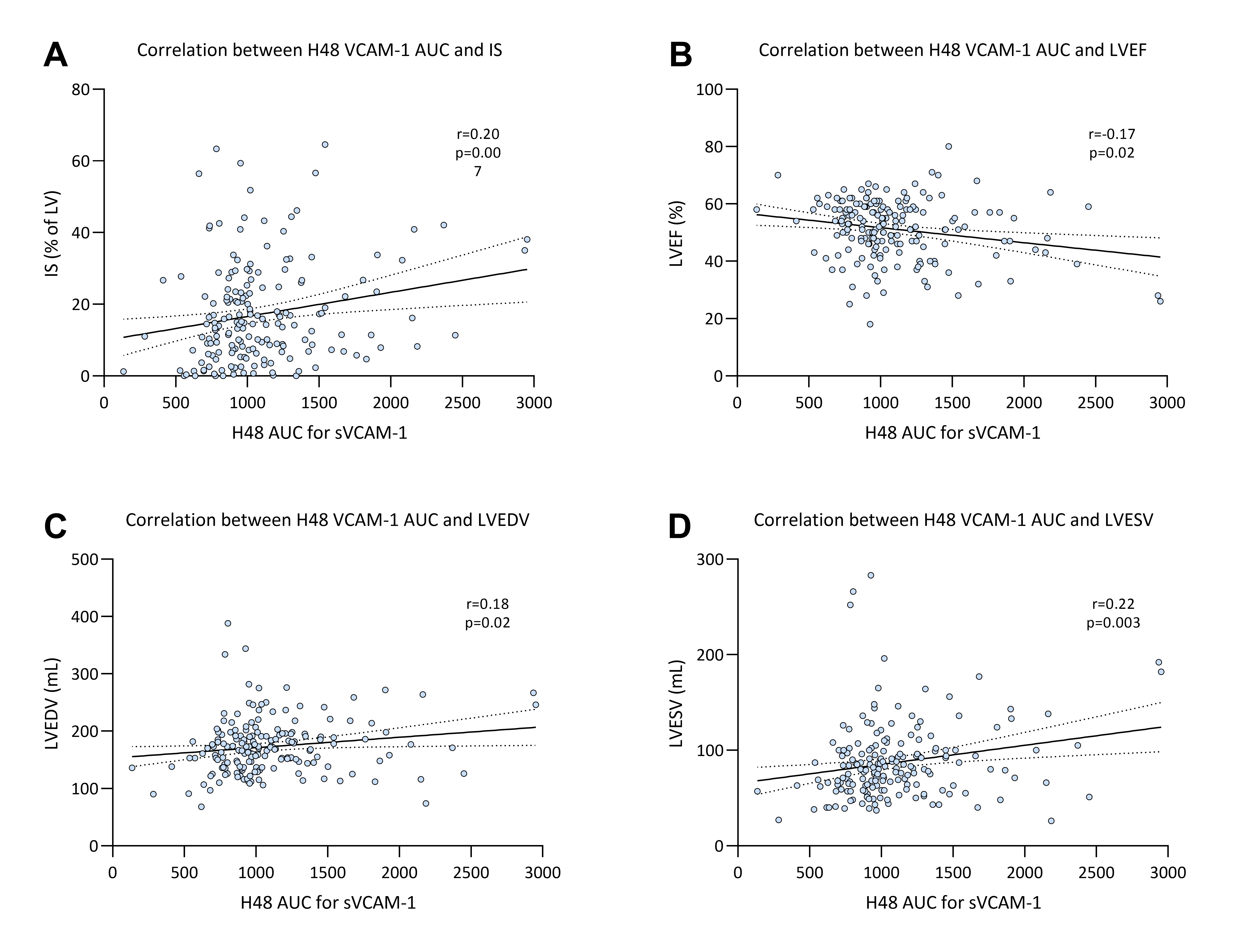

Supplement: Supplementary file 2 — Supporting information. [file IID3-9-493-s002.tif]
