# Supplementary material for: Kinetics and prognostic value of soluble VCAM‐1 in ST‐segment elevation myocardial infarction patients
Source: Immun Inflamm Dis. 2021 Feb 8;9(2):493–501. doi: 10.1002/iid3.409 (PMC8127550; doi:10.1002/iid3.409)
Supplement: Supplementary file 3 — Supporting information. [file IID3-9-493-s001.docx]

**Supplemental Figure legend :**

**Supplemental figure 1.**

**A.** Comparison of soluble VCAM-1 (sVCAM-1) kinetics in ST-segment elevation myocardial infarction (STEMI) patients within the first month (n=251 patients) according to **(A)** infarct size (≤25% vs. >25%), **(B)** MI topography (Anterior vs. non Anterior myocardial infarction) and **(C)** troponin peak quartiles (n=251).

**Supplemental figure 2.**

Correlation between H48 AUC and **(A)** infarct size (% of left ventricle), **(B)** left ventricular ejection fraction, **(C)** left ventricular end-diastolic volume and **(D)** left ventricular end-systolic volume in patients with STEMI measured with cardiac magnetic resonance at 1 month (n=251).

**Supplemental Table legend:**

**Suppplemental table 1.**

**Administered medication data of the study population (%).**

| **In-hospital treatment** | |
| --- | --- |
| Unfractionated heparin | 46.6% |
| Low molecular weight heparins | 43.8% |
| Glycoprotein IIb/IIIa inhibitors | 8.1% |
| Morphine | 39.6% |
| **Treatment at discharge** | |
| Aspirin | 97.7% |
| Thienopyridine | 98.1% |
| Betablockers | 95.3% |
| ACE inhibitors | 95.8% |
| Statins | 91.5% |
